# Heparan Sulfate Proteoglycans May Promote or Inhibit Cancer Progression by Interacting with Integrins and Affecting Cell Migration

**DOI:** 10.1155/2015/453801

**Published:** 2015-10-19

**Authors:** Mariana A. Soares, Felipe C. O. B. Teixeira, Miguel Fontes, Ana Lúcia Arêas, Marcelo G. Leal, Mauro S. G. Pavão, Mariana P. Stelling

**Affiliations:** ^1^Instituto de Bioquímica Médica, Universidade Federal do Rio de Janeiro, 21941-913 Rio de Janeiro, RJ, Brazil; ^2^Serviço de Patologia, Hospital Naval Marcílio Dias, 20725-090 Rio de Janeiro, RJ, Brazil; ^3^Hospital Naval Marcílio Dias, Instituto de Pesquisas Biomédicas, 20725-090 Rio de Janeiro, RJ, Brazil

## Abstract

The metastatic disease is one of the main consequences of tumor progression, being responsible for most cancer-related deaths worldwide. This review intends to present and discuss data on the relationship between integrins and heparan sulfate proteoglycans in health and cancer progression. Integrins are a family of cell surface transmembrane receptors, responsible for cell-matrix and cell-cell adhesion. Integrins' main functions include cell adhesion, migration, and survival. Heparan sulfate proteoglycans (HSPGs) are cell surface molecules that play important roles as cell receptors, cofactors, and overall direct or indirect contributors to cell organization. Both molecules can act in conjunction to modulate cell behavior and affect malignancy. In this review, we will discuss the different contexts in which various integrins, such as *α*5, *α*V, *β*1, and *β*3, interact with HSPGs species, such as syndecans and perlecans, affecting tissue homeostasis.

## 1. Introduction

Metastasis is the ultimate result of cancer progression. There are several factors involved in the establishment of a metastatic site. These factors may be produced by cancer cells or by other cell types upon stimulation by a tumor. This review intends to present and discuss data on the relationship between integrins and heparan sulfate proteoglycans (HSPGs) in physiological conditions and during cancer progression. These two classes of molecules are deeply involved in cancer progression and can be found on the cell surface and the extracellular matrix (HSPGs only). We will focus on the mechanisms involving direct or indirect interaction between integrins and HSPGs, leading to altered cell behavior, such as cell adhesion, spreading, and cytoskeleton organization.

## 2. Integrins' Functions

Integrins are a family of cell surface transmembrane receptors, responsible for cell-ECM and cell-cell adhesion [1–3]. Due to their functions, integrins are considered fundamental for multicellular organism development. They have been expressed since early metazoans, although gene sequences may differ from group to group [4, 5]. Integrin functions by promoting cell adhesion, connecting the intra- with the extracellular space, leading to cytoskeleton arrangement, cell survival, differentiation, and growth [6–8]. These functions are relevant in embryo development and wound healing, as well as in various pathologies. Integrins are the main components of adhesion force generation, important for mesenchymal-like migration and collective migration, both relevant in cancer [9].

They are composed of two subunits, *α* and *β*. *α* subunit has eighteen isoforms, with molecular weights ranging from 120 to 180 kDa, while *β* subunit has eight isoforms ranging from 90 to 110 kDa [2]. Both subunits have only one transmembrane segment [2]. Different combinations of *α* and *β* subunits provide different affinities for ECM molecules. Each integrin dimer binds to different substrates; however, binding may be redundant among dimers. In the following lines, we will present the *β* subunits mentioned in this review.

*β*1 integrin pairs with 12 *α* subunits. They virtually occur in all vertebrate cells. *β*1 knockout mice are not viable because the embryo cannot perform implantation in the uterine wall. *β*2 integrin pairs with 4 *α* subunits. They only occur on white blood cells and are responsible for cell-cell interactions. *β*3 integrin is found on blood platelets and other cells. *β*4 integrins are major components of hemidesmosomes and their interaction with keratin filaments is relevant for cell-ECM adhesion [10, 11].

Integrin ligands are comprised of laminins, collagens, and the RGD motif, present in fibronectin and other proteins. Integrins interact with many other molecules on the cell surface, integrating intra- and extracellular compartments [12]. When binding to the extracellular matrix (ECM) for migration purposes, integrins cluster, forming a focal adhesion site, while when no clustering occurs, it is usually for activation of intracellular signaling. Finally, integrin trafficking is the main regulatory process of integrin availability on the cell surface [13].

Integrins present different activation states. Divalent cations affect integrins affinity and specificity; a balance between calcium, zinc, magnesium, and manganese may modulate integrin binding to its substrate [14–17]. Among the divalent cations, manganese has the most extreme modulating effect on integrin affinity for its substrates [14, 16]. Magnesium also activates integrins, while zinc will keep integrins in an inactive state.

Inside-out integrin activation is relevant for defense responses, especially when immune cells must bind to the endothelium or reach damaged areas during an infection or inflammation event [18–20]. Finally, integrins are also relevant in cell survival; lack of contact with the ECM leads to cell death [8]. Epithelial cells may have a different relationship than stromal cells as they differ in cell-cell and cell-ECM binding.

## 3. Heparan Sulfate Proteoglycans and Integrins

HSPGs play important roles during development; there are many examples of their ability to regulate cell growth, angiogenesis, tumor development, and other events. The average heparan sulfate (HS) chain is 50–200 repeating disaccharide units in length and is typically responsible for the majority of HSPGs functions, including protein-binding activity [21]. Different cell types express the same type of proteoglycan; however, these core proteins may present structurally different HS chains [22]; this suggests a finely regulated tissue-specific synthesis. Posttranscriptional and posttranslational modifications also influence proteoglycan variability [23]. Protein binding is usually mediated by HS chains, mostly by clustering basic amino acid residues with negatively charged regions of the glycan [24], but may also involve the proteoglycan core protein [25, 26]. Despite the fact that sulfate residues are largely responsible for the ionic interactions between HS and proteins (such as integrins and growth factors), hydrogen bonds and van der Waals interactions also play a significant role in HS-protein binding [27]. HS tridimensional conformation, defined by modifications during its biosynthesis, is a determinant feature of interactions between HSPGs and proteins [28]. ECM proteins, such as fibronectin, can synergistically bind integrins and syndecans and activate the cytoplasmic domain of this proteoglycan, controlling cell adhesion and motility by interacting with intracellular components [29, 30].

### 3.1. Syndecan-1

Syndecans are a family of transmembrane proteoglycans expressed throughout the organism [31]. All syndecans isoforms are regulated during development [32]. Many biological processes have been described as dependent on the interaction between syndecans and integrins. Their role in cell spreading, for example, is performed by exposing binding sites on fibronectin that can be recognized by integrins [33] or by modulation of integrin activation state [34].

Syndecan-1 is largely expressed in epithelia, contributing to the organization of adhesion molecules. This property has been shown in many works, whereas syndecan-1 influences integrin activation and cell arrangement.

Kato and colleagues have shown altered cell migration and reorganization after syndecan-1 loss; these changes could be associated with embryogenesis processes or even carcinoma development [35]. Studies on syndecan-1 role in cell adhesion by specific integrins show that this proteoglycan influences *α*2*β*1, *α*V*β*3, and *α*V*β*5 integrins binding to collagen, vitronectin, and vitronectin/fibronectin, respectively [36–39].

Finally, integrins *α*V*β*3 and *α*V*β*5 can also be activated by an inside-out system involving syndecan-1, via formation of a ternary complex: integrin-syndecan-insulin-like growth factor receptor, which leads to intracellular activation of the integrin by talin [40], promoting endothelial cell migration.

### 3.2. Syndecan-2

Syndecan-2 is not an integrin ligand, but it can interfere with its activation. In fibroblasts, syndecan-2 ectodomain binds to the protein tyrosine kinase phosphatase receptor, CD148, leading to an intracellular signal that induces *β*1 integrin-mediated cell adhesion [41]. Another study has shown that when syndecan-2 is shed from the endothelial cell membrane, it presents paracrine interactions with CD148, which leads to deactivation of *β*1 integrins, promoting an antiangiogenic effect [42]. Finally, it has been shown that syndecan-2 mediates adhesion to fibronectin in osteoblasts, its downregulation leads to reduced cell adhesion and spreading, and syndecan-2 downstream signaling molecule, ROCK, is also reduced in this context [43].

Overall, syndecan-2 indirect effect on integrin activation participates in the organization of different tissues; we will explore its role in tumor development in the following sections.

### 3.3. Syndecan-4

Syndecan-4 is an important component of focal adhesion and is involved in cytoskeletal reorganization. *β*1 integrin-mediated adhesion requires syndecan-4; nevertheless, there is no evidence of direct contact between these two molecules [44, 45]. This suggests a possible link with CD148, similar to syndecan-2 influence on cell adhesion [41]. The work by Chung and colleagues reinforces this link by revealing syndecan-4 interaction with CD148 as an important factor in the inhibition of T-cell activation [46].

Syndecan-4 has also a major role in regulating matrix structure and cell adhesion/migration during all stages of embryonic development and in most adult tissues. This phenomenon is strongly dependent on the interaction with *β*1 integrins, such as *α*5*β*1, promoting focal adhesion assembly [47]. This assembly requires integrin turnover by endocytosis, which enables cell-ECM contact during migration [48, 49]. Another example of syndecan-4 influence on *β*1 integrins is the regulation of matrix structure described by Vuoriluoto and colleagues, whereas they show that syndecan-4 inhibits *α*2*β*1 integrin-mediated collagen invasion [50]. Finally, Rønning and colleagues have shown that syndecan-4 cytoplasmic domain inhibits myogenesis, and its silencing during muscle differentiation leads to a higher expression of *β*1 integrin, possibly leading to the formation of focal adhesion [51]. Interestingly, when Carneiro and colleagues produced endothelial cell lines resistant to anoikis, these cells maintained *β*5 integrin levels but presented higher syndecan-4 expression [52].

All these lines of evidence indicate that syndecan-4 has important roles in cell migration, and, according to the developmental context, it may promote or inhibit cell adhesion/migration by mechanisms directly or indirectly associated with integrins.

### 3.4. Perlecan

Perlecan is ubiquitously expressed within the ECM and the basement membrane. This HSPG mediates cell signaling and controls cell differentiation, proliferation, and migration [21, 26]. Many developmental and homeostatic processes, like cartilage formation and wound healing, are dependent on its presence [53]. Perlecan knockout mice are not viable, resulting in early neonatal death due to abnormalities in ECM organization [54].

In brain infarcts, endorepellin, also known as perlecan domain V, has proangiogenic activity in brain microvasculature when in combination with *α*5*β*1 integrins; this integrin dimer acts as a receptor for endorepellin and stimulates angiogenesis [55]. When endorepellin binds to *α*2*β*1 integrins on endothelial cells, it blocks cell migration and angiogenesis by disassembling actin-stress fibers and focal adhesion [56]; this could be a control mechanism to avoid exaggerated angiogenesis. Endorepellin specific activity on endothelial cells was recently explained by the need of simultaneous expression of *α*2*β*1 integrins and VEGFR2 found, so far, only in this cell type [57].

### 3.5. Agrin

Agrin is a multidomain ECM HSPG that was first discovered in neuromuscular junctions and other healthy tissues. It is widely expressed during development and plays a key role in the formation, maintenance, and regeneration of neuromuscular junctions [58]. It is known that *α*V and *β*1 integrins can act as receptors for agrin in muscle cells [59].

### 3.6. Collagen XVIII

Collagen XVIII is another HSPG with structural features of both collagens and proteoglycans [60]. Its C-terminal fraction, endostatin, interacts with *α*V*β*3 and *α*5*β*1 integrins, preventing endothelial cell migration and angiogenesis [61, 62].

These are examples of how HSPGs play key roles in integrin interaction with the ECM. Nowadays, many efforts are being made towards the elucidation of these interactions in order to develop better treatments to many diseases and malfunctions, especially cancer progression.

## 4. Heparan Sulfate Proteoglycans and Integrins in Cancer

HSPGs and integrins play important roles in cancer development. In this topic, we will describe the interactions between HSPGs and integrin and their effect on cancer progression.

### 4.1. Syndecan-1

Syndecans are one of the best portrayed HSPGs in studies on integrins and their engagement in cancer progression. Various interactions between these two classes of molecules modulate cell behavior in response to different signals [21, 63, 64]. Syndecan-1 association with integrins seems to generally induce tumor cell spreading and invasion, especially via interaction of its extracellular domain with *α*V*β*3 and *α*V*β*5 integrins [37, 38, 65]. Lines of evidence for these activities are described in the following lines.

MDA-MB-231 human breast carcinoma cells express syndecan-1 and syndecan-4. The signaling pathway associated with cell spreading in these cells seems to be dependent on *α*V*β*3 integrin and syndecan-1, while syndecan-4 does not seem to be involved in this mechanism [37]. In addition, Beauvais and colleagues have shown that syndecan-1 ectodomain is specifically relevant for *α*V*β*3 integrin binding to vitronectin in both MDA-MB-231 and MDA-MB-435 cell lines [38]. Syndecan-1 core protein and its complete form are not enough to establish adhesion sites on a collagen substrate by themselves; however, if this proteoglycan is presented in conjunction with *α*2*β*1 integrins in MDA-MB-231 cells, adhesion is possible, and HS chains are mandatory for this interaction [66]. All these facts highlight the importance of syndecan-1 ectodomain in pathologic cell behavior.

Indirect interactions between syndecan-1 and integrins have also been described, such as the one between *α*6*β*4 integrin and syndecan-1, an interaction mediated by human epidermal growth factor receptor 2 (HER2) that leads to tumor cell survival* in vitro* [67].

Syndecan-1 also affects other aspects of tumor progression, such as angiogenesis promotion during tumorigenesis. The work by Beauvais and colleagues shows that synstatin, a peptide derived from syndecan-1 active core protein, has antiangiogenic properties* in vivo* and* in vitro*, in addition to decreasing mammary carcinoma formation in nude mice. In this context, *α*V*β*3 and *α*V*β*5 integrins are important to regulate angiogenesis [68], and, while syndecan-1 is necessary to regulate both integrins during angiogenesis and tumorigenesis, synstatin can cause the outbreak of this interaction [65].

Finally, syndecan-1 can also indirectly interfere with integrin by increasing integrin-ECM binding or by amplification of integrin signaling [37]. The work by Yang and colleagues shows that human myocardial fibroblasts secrete a fibronectin-rich ECM, which presents organized, parallel, fiber architecture. This fiber organization is dependent on syndecan-1 presence and is fundamental for the attachment and migration of breast carcinoma cells. This attachment probably occurs because this proteoglycan regulates the activity of several integrins, promoting fibronectin matrix assembly [69].

### 4.2. Syndecan-2

Many reports present syndecan-2 as an inhibitor of metastatic behavior. Munesue and colleagues have shown that low metastatic clones of Lewis lung carcinoma (LLC) cells present high syndecan-2 expression, while the highly metastatic clone does not. Induction of syndecan-2 expression in the highly metastatic clone mimics the low metastatic clone behavior, with the formation of actin-stress fibers mediated by *α*5*β*1 integrin that, ultimately, will reflect on low invasive capacity [70, 71].

Syndecan-2 shedding has also an antiangiogenic effect in endothelium. CD148 interacts with shed syndecan-2 in endothelial cells, causing changes in *β*1 integrin activation state, which leads to angiogenesis inhibition, affecting tumor growth [72]. This fact could be taken into account as an important way to develop novel therapies for diseases strongly dependent on angiogenesis for progression.

On the other hand, syndecan-2 may also promote invasiveness, as seen in MDA-MB-231 cells, whereas this proteoglycan has an important role in cell spreading and adhesion, leading to invasiveness and preserving a malignant phenotype, dependent on Rho GTPases, which regulates the actin cytoskeleton [73].

### 4.3. Syndecan-4

Syndecan-4 physiological role in focal adhesion formation can also be translated into tumor progression. Many reports have shown its importance for tumor cell survival, adhesion, and migration in the various conditions faced by a tumor cell during cancer progression, such as the ability to bind to the endothelium or thrive in hypoxic conditions.

Syndecan-4 phosphorylation was found to have an important role in the control of integrin recycling. This proteoglycan can control *α*V*β*3 integrin trafficking to the plasma membrane, promoting sustained focal adhesion in healthy mouse cells. The essential molecules in this process, as well as integrin recycling events and integrin expression changes, are found in processes like tumor invasion, demonstrating a route that can be further studied in cancer progression [74–77].

Syndecan-4 has also been associated with the metastatic phenotype; analyses of renal cell carcinoma samples and the highly metastatic tumor cell line KP1 have revealed an association between aggressive phenotype and high expression of tissue transglutaminase (TG2) and syndecan-4. This fact can be associated with syndecan-4 and *α*5*β*1 integrin interactions [78, 79].

It was recently discovered that the endothelial surface molecule Thy-1 (CD90) is important for B16/F10 melanoma cells adhesion to endothelium via *α*V*β*3 integrin, favoring metastasis in an* in vivo* model [80]. Likewise, syndecan-4 promotes A375 melanoma cells binding to the endothelium by participating of a ternary complex with *α*5*β*1 integrins and Thy-1. This complex promotes a strong interaction between the tumor cell and the endothelium, which is suitable for downstream mechanosignaling [81].

Cancer cells change their expression profile when challenged in hypoxic conditions. Koike and colleagues have shown that hypoxic human colon cancer cells remarkably overexpress syndecan-4 and *α*5 integrin, which are important cell-adhesion molecules involved in the enhanced adhesion of cancer cells to fibronectin [82].

Overall, syndecan-4 is a versatile molecule regarding tumor progression and more studies on its roles in cell physiology and the changes that accompany an invasive phenotype are needed for further advances in this field.

### 4.4. Perlecan

High expression of perlecan was found in some carcinomas, suggesting its involvement in disease progression [83]. Perlecan role in human squamous cell carcinoma progression may be due to recognition by its two receptors, *α*-dystroglycan and *β*1 integrin. This association happens not only in physiological conditions [84], but also in invasive carcinoma, epithelial dysplasia, and carcinoma* in situ* [3]. Ameloblastoma presents high expression of *α*-dystroglycan and *β*1 integrin, indicating the importance of perlecan signaling in this type of cancer as well [85].

Endorepellin has potent antiangiogenic activity [26, 86]. It was shown that antiangiogenic and antitumor growth effects of endorepellin occur due to its interaction with *α*2*β*1 integrin [87]. It was also observed that endorepellin needs *α*2*β*1 integrin and VEGFR2 (vascular endothelial growth factor receptor 2) to promote angiostatic activity in human umbilical vein endothelial cells (HUVECs) and porcine aortic endothelial (PAE) cells [88]. These studies may be useful in the development of strategies to delay cancer progression, since perlecan and endorepellin were shown to affect tumor angiogenesis.

### 4.5. Agrin

Agrin is highly expressed in carcinomas such as hepatocellular carcinoma (HCC) and cholangiocarcinoma [89–92]. It is known that agrin is capable of interacting with *α*V and *β*1 integrins [59]. Hepatocellular carcinoma exhibits *α*V integrin and agrin near vessels and bile ducts, suggesting that both molecules may promote cancer progression by increasing angiogenesis [89, 93].

### 4.6. Neuropilin-1

Neuropilin-1 (NRP-1) is a membrane bound HSPG that is expressed in normal tissues and in tumors like glioma, breast, colon, and pancreas. In addition, it is expressed in tumor vessels, being usually overexpressed in invasive cancers in comparison to neighboring healthy tissue. Overall, NRP-1 can be related to cancer aggressiveness [21, 94, 95]. NRP-1 is known to interact with VEGF receptor being a VEGF-dependent functional regulator [94, 95]. The presence of NRP-1 and integrins correlates with a more aggressive melanoma [96]. Melanomas which express NRP-1 become more aggressive due to the activation of *α*V integrin, a marker molecule in the conversion of melanoma cells to a metastatic phenotype [96]. Ruffini and colleagues found that *α*V*β*5 integrin was involved in the transformation of cells expressing NRP-1. They have also identified a mechanism in which *α*V*β*5 integrin inhibitor affects melanoma progression by delaying angiogenesis [96]. In this same study, it was shown that *α*V*β*3 integrin promoted ECM invasion in the presence of VEGFR-2 in NRP-1-positive melanoma cells [96].

NRP-1 expression is increased by a glycoprotein named transmembrane NMB (GPNMB), which is known to promote malignant phenotype in breast cancer [97, 98]. GPNMB is able to bind *α*5*β*1 integrin, which activates a signaling pathway related to invasion and metastasis. Thus, GPNMB and NRP-1 must have an important role in mammary tumor growth and metastasis mediated by *α*5*β*1 integrin [98].

### 4.7. Betaglycan

Betaglycan, also known as TGF-*β* receptor type III (T*β*RIII), is a transmembrane proteoglycan that functions as a coreceptor for TGF-*β* [99, 100]. It possesses antitumoral activity by reducing cell motility and survival. In human breast cancer, T*β*RIII alters *α*5 integrin localization to sites of adhesion and the reduction of T*β*RIII gene expression was found to reduce overall survival in breast cancer patients. T*β*RIII suppresses cancer progression by stabilizing the ECM and by accumulating *α*5*β*1 integrin in its activated state; therefore, T*β*RIII decreased expression could disrupt ECM structure and influence *α*5 integrin localization, promoting cancer progression by enhancing cell motility and invasion [99].

In another study, it was shown that T*β*RIII knockdown decreases migratory and invasive characteristics of mesenchymal-stem-like (MSL)/triple negative breast cancer (TNBC) cells. This study shows that T*β*RIII knockdown is necessary to enhance *α*2 integrin expression, which leads to a decrease in migration and invasion of MSL/TNBC [101].

## 5. Closing Remarks

Integrins and heparan sulfate proteoglycans are versatile molecules that may present different functions according to the environment. Research on these molecules as agents in tumor progression is fundamental and brings to light the intricate, complex relationships occurring at cellular and subcellular levels. By analyzing HSPGs-integrin conjunct function in different types of cancer, we might be able to develop treatments based on analog molecules or develop prognostic techniques that may aid in patient treatment design. We also believe it is paramount to consider studies on other glycosaminoglycans, such as chondroitin sulfate, which may be of importance for indirect interactions with integrins.

In conclusion, we believe that as knowledge on how integrins and GAGs interact grows, our chances in succeeding to unveil mechanisms of tumor progression inhibition will be greater.

## References

[1] Hynes R. O. (1987). Integrins: a family of cell surface receptors. *Cell*.

[2] Hynes R. O. (1992). Integrins: versatility, modulation, and signaling in cell adhesion. *Cell*.

[3] Tamkun J. W., DeSimone D. W., Fonda D. (1986). Structure of integrin, a glycoprotein involved in the transmembrane linkage between fibronectin and actin. *Cell*.

[4] Brower D. L., Brower S. M., Hayward D. C., Ball E. E. (1997). Molecular evolution of integrins: genes encoding integrin *β* subunits from a coral and a sponge. *Proceedings of the National Academy of Sciences of the United States of America*.

[5] Knack B. A., Iguchi A., Shinzato C., Hayward D. C., Ball E. E., Miller D. J. (2008). Unexpected diversity of cnidarian integrins: expression during coral gastrulation. *BMC Evolutionary Biology*.

[6] Nisticò P., Di Modugno F., Spada S., Bissell M. J. (2014). *β*1 and *β*4 integrins: from breast development to clinical practice. *Breast Cancer Research*.

[7] Cabodi S., Di Stefano P., Leal M. D. P. C. (2010). Integrins and signal transduction. *Advances in Experimental Medicine and Biology*.

[8] Iwamoto D. V., Calderwood D. A. (2015). Regulation of integrin-mediated adhesions. *Current Opinion in Cell Biology*.

[9] Schmidt S., Friedl P. (2010). Interstitial cell migration: integrin-dependent and alternative adhesion mechanisms. *Cell and Tissue Research*.

[10] Seltmann K., Cheng F., Wiche G., Eriksson J. E., Magin T. M. (2015). Keratins stabilize hemidesmosomes through regulation of *β*4-integrin turnover. *Journal of Investigative Dermatology*.

[11] Guo W., Pylayeva Y., Pepe A. (2006). *β*4 integrin amplifies ErbB2 signaling to promote mammary tumorigenesis. *Cell*.

[12] Humphries M. J., Travis M. A., Clark K., Mould A. P. (2004). Mechanisms of integration of cells and extracellular matrices by integrins. *Biochemical Society Transactions*.

[13] De Franceschi N., Hamidi H., Alanko J., Sahgal P., Ivaska J. (2015). Integrin traffic—the update. *Journal of Cell Science*.

[14] Gailit J., Ruoslahti E. (1988). Regulation of the fibronectin receptor affinity by divalent cations. *Journal of Biological Chemistry*.

[15] Grzesiak J. J., Davis G. E., Kirchhofer D., Pierschbacher M. D. (1992). Regulation of *α*2*β*1-mediated fibroblast migration on type I collagen by shifts in the concentrations of extracellular Mg^2+^ and Ca^2+^. *Journal of Cell Biology*.

[16] Kirchhofer D., Gailit J., Ruoslahti E., Grzesiak J., Pierschbacher M. D. (1990). Cation-dependent changes in the binding specificity of the platelet receptor GPIIb/IIIa. *Journal of Biological Chemistry*.

[17] Lymburner S., McLeod S., Purtzki M., Roskelley C., Xu Z. (2013). Zinc inhibits magnesium-dependent migration of human breast cancer MDA-MB-231 cells on fibronectin. *Journal of Nutritional Biochemistry*.

[18] Ye F., Kim C., Ginsberg M. H. (2011). Molecular mechanism of inside-out integrin regulation. *Journal of Thrombosis and Haemostasis*.

[19] Laudanna C., Bolomini-Vittori M. (2009). Integrin activation in the immune system. *Wiley Interdisciplinary Reviews: Systems Biology and Medicine*.

[20] Kinashi T. (2012). Overview of integrin signaling in the immune system. *Methods in Molecular Biology*.

[21] Sarrazin S., Lamanna W. C., Esko J. D. (2011). Heparan sulfate proteoglycans. *Cold Spring Harbor Perspectives in Biology*.

[22] Okolicsanyi R. K., Griffiths L. R., Haupt L. M. (2014). Mesenchymal stem cells, neural lineage potential, heparan sulfate proteoglycans and the matrix. *Developmental Biology*.

[23] Kim S.-H., Turnbull J., Guimond S. (2011). Extracellular matrix and cell signalling: the dynamic cooperation of integrin, proteoglycan and growth factor receptor. *Journal of Endocrinology*.

[24] Cardin A. D., Weintraub H. J. R. (1989). Molecular modeling of protein-glycosaminoglycan interactions. *Arteriosclerosis, Thrombosis, and Vascular Biology*.

[25] Kirkpatrick C. A., Knox S. M., Staatz W. D., Fox B., Lercher D. M., Selleck S. B. (2006). The function of a *Drosophila* glypican does not depend entirely on heparan sulfate modification. *Developmental Biology*.

[26] Whitelock J. M., Melrose J., Iozzo R. V. (2008). Diverse cell signaling events modulated by Perlecan. *Biochemistry*.

[27] Faham S., Hileman R. E., Fromm J. R., Linhardt R. J., Rees D. C. (1996). Heparin structure and interactions with basic fibroblast growth factor. *Science*.

[28] Skidmore M. A., Guimond S. E., Rudd T. R., Fernig D. G., Turnbull J. E., Yates E. A. (2008). The activities of heparan sulfate and its analogue heparin are dictated by biosynthesis, sequence, and conformation. *Connective Tissue Research*.

[29] Alexopoulou A. N., Multhaupt H. A. B., Couchman J. R. (2007). Syndecans in wound healing, inflammation and vascular biology. *International Journal of Biochemistry and Cell Biology*.

[30] Mahalingam Y., Gallagher J. T., Couchman J. R. (2007). Cellular adhesion responses to the heparin-binding (HepII) domain of fibronectin require heparan sulfate with specific properties. *Journal of Biological Chemistry*.

[31] Saunders S., Jalkanen M., O'Farrell S., Bernfield M. (1989). Molecular cloning of syndecan, an integrated membrane proteoglycan. *Journal of Cell Biology*.

[32] Rapraeger A. C. (2001). Molecular interactions of syndecans during development. *Seminars in Cell and Developmental Biology*.

[33] Khan M. Y., Jaikaria N. S., Frenz D. A., Villanueva G., Newman S. A. (1988). Structural changes in the NH2-terminal domain of fibronectin upon interaction with heparin. Relationship to matrix-driven translocation. *The Journal of Biological Chemistry*.

[34] Iba K., Albrechtsen R., Gilpin B. (2000). The cysteine-rich domain of human ADAM 12 supports cell adhesion through syndecans and triggers signaling events that lead to beta1 integrin-dependent cell spreading. *Journal of Cell Biology*.

[35] Kato M., Saunders S., Nguyen H., Bernfield M. (1995). Loss of cell surface syndecan-1 causes epithelia to transform into anchorage-independent mesenchyme-like cells. *Molecular Biology of the Cell*.

[36] Ishikawa T., Kramer R. H. (2010). Sdc1 negatively modulates carcinoma cell motility and invasion. *Experimental Cell Research*.

[37] Beauvais D. M., Rapraeger A. C. (2003). Syndecan-1-mediated cell spreading requires signaling by *α*v*β*3 integrins in human breast carcinoma cells. *Experimental Cell Research*.

[38] Beauvais D. M., Burbach B. J., Rapraeger A. C. (2004). The syndecan-1 ectodomain regulates *α*
_*v*_
*β*
_3_ integrin activity in human mammary carcinoma cells. *The Journal of Cell Biology*.

[39] McQuade K. J., Beauvais D. M., Burbach B. J., Rapraeger A. C. (2006). Syndecan-1 regulates *α*
_v_
*β*5 integrin activity in B82L fibroblasts. *Journal of Cell Science*.

[40] Beauvais D. M., Rapraeger A. C. (2010). Syndecan-1 couples the insulin-like growth factor-1 receptor to inside-out integrin activation. *Journal of Cell Science*.

[41] Whiteford J. R., Xian X., Chaussade C., Vanhaesebroeck B., Nourshargh S., Couchman J. R. (2011). Syndecan-2 is a novel ligand for the protein tyrosine phosphatase receptor CD148. *Molecular Biology of the Cell*.

[42] de Rossi G., Evans A. R., Kay E. (2014). Shed syndecan-2 inhibits angiogenesis. *Journal of Cell Science*.

[43] Wang Z., Telci D., Griffin M. (2011). Importance of syndecan-4 and syndecan -2 in osteoblast cell adhesion and survival mediated by a tissue transglutaminase-fibronectin complex. *Experimental Cell Research*.

[44] Whiteford J. R., Behrends V., Kirby H., Kusche-Gullberg M., Muramatsu T., Couchman J. R. (2007). Syndecans promote integrin-mediated adhesion of mesenchymal cells in two distinct pathways. *Experimental Cell Research*.

[45] Whiteford J. R., Couchman J. R. (2006). A conserved NXIP motif is required for cell adhesion properties of the syndecan-4 ectodomain. *The Journal of Biological Chemistry*.

[46] Chung J.-S., Cruz P. D., Ariizumi K. (2011). Inhibition of T-cell activation by syndecan-4 is mediated by CD148 through protein tyrosine phosphatase activity. *European Journal of Immunology*.

[47] Woods A., Couchman J. R. (2001). Syndecan-4 and focal adhesion function. *Current Opinion in Cell Biology*.

[48] Bass M. D., Williamson R. C., Nunan R. D. (2011). A syndecan-4 hair trigger initiates wound healing through caveolin- and RhoG-regulated integrin endocytosis. *Developmental Cell*.

[49] Elfenbein A., Rhodes J. M., Meller J., Schwartz M. A., Matsuda M., Simons M. (2009). Suppression of RhoG activity is mediated by a syndecan 4-synectin-RhoGDI1 complex and is reversed by PKC*α* in a Rac1 activation pathway. *The Journal of Cell Biology*.

[50] Vuoriluoto K., Högnäs G., Meller P., Lehti K., Ivaska J. (2011). Syndecan-1 and -4 differentially regulate oncogenic K-ras dependent cell invasion into collagen through *α*2*β*1 integrin and MT1-MMP. *Matrix Biology*.

[51] Rønning S. B., Carlson C. R., Stang E., Kolset S. O., Hollung K., Pedersen M. E. (2015). Syndecan-4 regulates muscle differentiation and is internalized from the plasma membrane during myogenesis. *PLoS ONE*.

[52] Carneiro B. R., Pernambuco Filho P. C. A., de Sousa Mesquita A. P. (2014). Acquisition of anoikis resistance up-regulates syndecan-4 expression in endothelial cells. *PLoS ONE*.

[53] Knox S. M., Whitelock J. M. (2006). Perlecan: how does one molecule do so many things?. *Cellular and Molecular Life Sciences*.

[54] Bülow H. E., Hobert O. (2006). The molecular diversity of glycosaminoglycans shapes animal development. *Annual Review of Cell and Developmental Biology*.

[55] Lee B., Clarke D., Al Ahmad A. (2011). Perlecan domain V is neuroprotective and proangiogenic following ischemic stroke in rodents. *The Journal of Clinical Investigation*.

[56] Bix G., Fu J., Gonzalez E. M. (2004). Endorepellin causes endothelial cell disassembly of actin cytoskeleton and focal adhesions through *α*2*βα*1 integrin. *Journal of Cell Biology*.

[57] Goyal A., Pal N., Concannon M. (2011). Endorepellin, the angiostatic module of perlecan, interacts with both the *α*2*β*1 integrin and vascular endothelial growth factor receptor 2 (VEGFR2): a dual receptor antagonism. *The Journal of Biological Chemistry*.

[58] Bezakova G., Ruegg M. A. (2003). New insights into the roles of agrin. *Nature Reviews Molecular Cell Biology*.

[59] Martin P. T., Sanes J. R. (1997). Integrins mediate adhesion to agrin and modulate agrin signaling. *Development*.

[60] Seppinen L., Pihlajaniemi T. (2011). The multiple functions of collagen XVIII in development and disease. *Matrix Biology*.

[61] Rehn M., Veikkola T., Kukk-Valdre E. (2001). Interaction of endostatin with integrins implicated in angiogenesis. *Proceedings of the National Academy of Sciences of the United States of America*.

[62] Sudhakar A., Sugimoto H., Yang C., Lively J., Zeisberg M., Kalluri R. (2003). Human tumstatin and human endostatin exhibit distinct antiangiogenic activities mediated by *α*v*β* and *α*5*β*1 integrins. *Proceedings of the National Academy of Sciences of the United States of America*.

[63] Morgan M. R., Humphries M. J., Bass M. D. (2007). Synergistic control of cell adhesion by integrins and syndecans. *Nature Reviews Molecular Cell Biology*.

[64] Roper J. A., Williamson R. C., Bass M. D. (2012). Syndecan and integrin interactomes: large complexes in small spaces. *Current Opinion in Structural Biology*.

[65] Beauvais D. M., Ell B. J., McWhorter A. R., Rapraeger A. C. (2009). Syndecan-1 regulates *α*v*β*3 and *α*v*β*5 integrin activation during angiogenesis and is blocked by synstatin, a novel peptide inhibitor. *Journal of Experimental Medicine*.

[66] Vuoriluoto K., Jokinen J., Kallio K., Salmivirta M., Heino J., Ivaska J. (2008). Syndecan-1 supports integrin *α*2*β*1-mediated adhesion to collagen. *Experimental Cell Research*.

[67] Wang H., Leavitt L., Ramaswamy R., Rapraeger A. C. (2010). Interaction of syndecan and *α*6*β*4 integrin cytoplasmic domains. *The Journal of Biological Chemistry*.

[68] Friedlander M., Brooks P. C., Shaffer R. W., Kincaid C. M., Varner J. A., Cheresh D. A. (1995). Definition of two angiogenic pathways by distinct *α*
_*v*_ integrins. *Science*.

[69] Yang N., Mosher R., Seo S., Beebe D., Friedl A. (2011). Syndecan-1 in breast cancer stroma fibroblasts regulates extracellular matrix fiber organization and carcinoma cell motility. *American Journal of Pathology*.

[70] Munesue S., Kusano Y., Oguri K. (2002). The role of syndecan-2 in regulation of actin-cytoskeletal organization of Lewis lung carcinoma-derived metastatic clones. *Biochemical Journal*.

[71] Kusano Y., Yoshitomi Y., Munesue S., Okayama M., Oguri K. (2004). Cooperation of syndecan-2 and syndecan-4 among cell surface heparan sulfate proteoglycans in the actin cytoskeletal organization of Lewis lung carcinoma cells. *Journal of Biochemistry*.

[72] De Rossi G., Evans A. R., Kay E. (2014). Shed syndecan-2 inhibits angiogenesis. *Journal of Cell Science*.

[73] Lim H. C., Couchman J. R. (2014). Syndecan-2 regulation of morphology in breast carcinoma cells is dependent on RhoGTPases. *Biochimica et Biophysica Acta—General Subjects*.

[74] Morgan M. R., Hamidi H., Bass M. D., Warwood S., Ballestrem C., Humphries M. J. (2013). Syndecan-4 phosphorylation is a control point for integrin recycling. *Developmental Cell*.

[75] Caswell P. T., Vadrevu S., Norman J. C. (2009). Integrins: masters and slaves of endocytic transport. *Nature Reviews Molecular Cell Biology*.

[76] Muralidharan-Chari V., Hoover H., Clancy J. (2009). ADP-Ribosylation factor 6 regulates tumorigenic and invasive properties in vivo. *Cancer Research*.

[77] Playford M. P., Schaller M. D. (2004). The interplay between Src and integrins in normal and tumor biology. *Oncogene*.

[78] Erdem M., Erdem S., Sanli O. (2014). Up-regulation of TGM2 with ITGB1 and SDC4 is important in the development and metastasis of renal cell carcinoma. *Urologic Oncology: Seminars and Original Investigations*.

[79] Wang Z., Griffin M. (2013). The role of TG2 in regulating S100A4-mediated mammary tumour cell migration. *PLoS ONE*.

[80] Schubert K., Gutknecht D., Köberle M., Anderegg U., Saalbach A. (2013). Melanoma cells use thy-1 (CD90) on endothelial cells for metastasis formation. *American Journal of Pathology*.

[81] Fiore V. F., Ju L., Chen Y., Zhu C., Barker T. H. (2014). Dynamic catch of a Thy-1-*α*
_5_
*β*
_1_+syndecan-4 trimolecular complex. *Nature Communications*.

[82] Koike T., Kimura N., Miyazaki K. (2004). Hypoxia induces adhesion molecules on cancer cells: a missing link between Warburg effect and induction of selectin-ligand carbohydrates. *Proceedings of the National Academy of Sciences of the United States of America*.

[83] Ahsan M. S., Yamazaki M., Maruyama S. (2011). Differential expression of perlecan receptors, *α*-dystroglycan and integrin *β*1, before and after invasion of oral squamous cell carcinoma. *Journal of Oral Pathology and Medicine*.

[84] Henry M. D., Satz J. S., Brakebusch C. (2001). Distinct roles for dystroglycan, beta1 integrin and perlecan in cell surface laminin organization. *Journal of Cell Science*.

[85] Ida-Yonemochi H., Ahsan M. S., Saku T. (2011). Differential expression profiles between *α*-dystroglycan and integrin *β*1 in ameloblastoma: two possible perlecan signalling pathways for cellular growth and differentiation. *Histopathology*.

[86] Mongiat M., Sweeney S. M., San Antonio J. D., Fu J., Iozzo R. V. (2003). Endorepellin, a novel inhibitor of angiogenesis derived from the C terminus of perlecan. *Journal of Biological Chemistry*.

[87] Woodall B. P., Nyström A., Iozzo R. A. (2008). Integrin *α*2*β*1 is the required receptor for endorepellin angiostatic activity. *The Journal of Biological Chemistry*.

[88] Goyal A., Pal N., Concannon M. (2011). Endorepellin, the angiostatic module of perlecan, interacts with both the *α*2*β*1 integrin and vascular endothelial growth factor receptor 2 (VEGFR2): a dual receptor antagonism. *The Journal of Biological Chemistry*.

[89] Tátrai P., Dudás J., Batmunkh E. (2006). Agrin, a novel basement membrane component in human and rat liver, accumulates in cirrhosis and hepatocellular carcinoma. *Laboratory Investigation*.

[90] Somorácz Á., Tátrai P., Horváth G. (2010). Agrin immunohistochemistry facilitates the determination of primary versus metastatic origin of liver carcinomas. *Human Pathology*.

[91] Batmunkh E., Tátrai P., Szabó E. (2007). Comparison of the expression of agrin, a basement membrane heparan sulfate proteoglycan, in cholangiocarcinoma and hepatocellular carcinoma. *Human Pathology*.

[92] Kawahara R., Granato D. C., Carnielli C. M. (2014). Agrin and perlecan mediate tumorigenic processes in oral squamous cell carcinoma. *PLoS ONE*.

[93] Trikha M., Zhou Z., Nemeth J. A. (2004). CNTO 95, a fully human monoclonal antibody that inhibits *α*v integrins, has antitumor and antiangiogenic activity in vivo. *International Journal of Cancer*.

[94] Wu H., Chen H., Pan D. (2014). Imaging integrin *α*v*β*3 and NRP-1positive gliomas with a novel fluorine-18 labeled RGD-ATWLPPR heterodimeric peptide probe. *Molecular Imaging and Biology*.

[95] Jubb A. M., Strickland L. A., Liu S. D., Mak J., Schmidt M., Koeppen H. (2012). Neuropilin-1 expression in cancer and development. *The Journal of Pathology*.

[96] Ruffini F., Graziani G., Levati L., Tentori L., D'Atri S., Lacal P. M. (2015). Cilengitide downmodulates invasiveness and vasculogenic mimicry of neuropilin 1 expressing melanoma cells through the inhibition of *α*v*β*5 integrin. *International Journal of Cancer*.

[97] Rose A. A. N., Annis M. G., Dong Z. (2010). ADAM10 releases a soluble form of the GPNMB/osteoactivin extracellular domain with angiogenic properties. *PLoS ONE*.

[98] Maric G., Annis M. G., Dong Z. (2015). GPNMB cooperates with neuropilin-1 to promote mammary tumor growth and engages integrin *α*
_5_
*β*
_1_ for efficient breast cancer metastasis. *Oncogene*.

[99] Mythreye K., Knelson E. H., Gatza C. E., Gatza M. L., Blobe G. C. (2013). T*β*RIII/*β*-arrestin2 regulates integrin *α*5*β*1 trafficking, function, and localization in epithelial cells. *Oncogene*.

[100] López-Casillas F., Wrana J. L., Massagué J. (1993). Betaglycan presents ligand to the TGF*β* signaling receptor. *Cell*.

[101] Jovanović B., Beeler J. S., Pickup M. W. (2014). Transforming growth factor beta receptor type III is a tumor promoter in mesenchymal-stem like triple negative breast cancer. *Breast Cancer Research*.

